# Psychometric properties of the Persian version of the electroconvulsive related anxiety questionnaire for psychiatric patients

**DOI:** 10.3389/fpsyg.2025.1472259

**Published:** 2025-02-18

**Authors:** Azadeh EghbalManesh, Abbas Ebadi, Mohammad Zoladl, Zahra Rasoul Zadeh Haghighi

**Affiliations:** ^1^Department of Nursing, Faculty of Nursing and Midwifery, Tehran Medical Science, Islamic Azad University, Tehran, Iran; ^2^Nursing Care Research Center, Clinical Sciences Institute, Baqiyatallah University of Medical Sciences, Tehran, Iran; ^3^Department of Nursing, Yasuj University of Medical Sciences, Yasuj, Iran; ^4^Yasuj University of Medical Sciences, Yasuj, Iran

**Keywords:** anxiety, electroconvulsive therapy, Persian version, psychiatric patients, psychometric properties

## Abstract

**Introduction:**

ECT is one of the most effective treatments for psychiatric patients. The results of several studies have reported ECT-related anxiety, which makes the patients ignore the advantages of the treatment and subsequently avoid it. Adopting an appropriate instrument to evaluate and manage this anxiety is so significant. Since there is no valid questionnaire to assess the patients' anxiety for ECT in Persian, we aimed to translate the EARQ into Persian and evaluate its validity and reliability in the present study.

**Methods:**

In this methodological study, 170 patients with MDD, BMD, and schizophrenia aged 20–73 were recruited through convenience sampling and completed the 17-item EARQ in 2023. We used all aspects of face, content, and construct validity for the questionnaire. McDonald's Omega was calculated for the domains and 17 items to assess the reliability of the questionnaire.

**Results:**

Exploratory and confirmatory factor analysis revealed three factors, death anxiety, physical concern, and mental concern. Confirmatory factor analysis indicated moderate fit indices to support the three domains: CMIN/DF = 4.8 (*p* < 0/05), RMSEA = 0.152, CFI = 0.92, and NFI = 0.91; GFI = 0.72. In our study, McDonald's Omega was 0.965 for death anxiety, 0.91 for physical concerns, 0.964 for brain concerns, and a total of 0.98.

**Conclusions:**

The EARQ has acceptable validity and reliability, so specialists can use it to assess patients' anxiety before ECT and, according to the score, use suitable interventions to eliminate it.

## 1 Introduction

ECT (Electroconvulsive Therapy), developed in the 1930s, is a medical therapy used to treat various mental illnesses, such as mood disorders, schizophrenia, catatonia, depression, and suicidal ideation (Buday et al., 2024). It has many side effects, including memory disorders, headaches, nausea, cardiovascular issues, lethargy, and fatigue, which patients and caregivers are concerned about (Sadock et al., 2015). In addition to patients, care providers are also worried about these side effects, particularly memory loss, since they significantly influence the quality of life (Kumar et al., 2016).

On the other hand, in Asian countries, there is often a lack of awareness, misinformation, and a negative image of ECT (Chanpattana et al., 2010). This treatment has also been the subject of controversy and terrifying media coverage. For example, in 1970, the famous film “One Flew Over the Cuckoo's Nest” not only showed this treatment as painful, harmful, and with little therapeutic benefit but also presented the nurses' faces as apathetic, which intensified the fear and anxiety of patients and their caregivers about this treatment (Boschma, 2015).

According to repeated observations, ECT-related anxiety affects 14% to 75% of patients before, during, and after treatment (Obbels et al., 2017). This prevalence is comparable to patients' anxiety, which affects 11%−80% of patients before surgery (Maranets and Kain, 2000). Anxiety in patients causes them to ignore the advantages of the treatment and subsequently avoid it. According to Ayd Jr (1956), anxiety is the most disturbing psychological complication of ECT. Therefore, it is important to identify anxiety in individuals at the very beginning to perform the necessary psychotherapeutic and supportive interventions (Edmonson, 2020).

Obbels et al. (2020) designed and developed the ERAQ (Electroconvulsive Related Anxiety Questionnaire) for this purpose, found it easy to administer and confirmed the validity and reliability of the English and Dutch versions. It includes 17 items that describe the patient's feelings about ECT-related issues. On a 4-point Likert scale, the patients rated the scale from never (1) to too much (4). The overall score of this questionnaire ranges from 17 to 68. The higher the patient's score is, the more anxiety they experience (Obbels et al., 2020).

To the best of our knowledge, since there is no specific Persian questionnaire to assess ECT-related anxiety, the researcher in this study decided to translate it and evaluate its validity and reliability. Additionally, Specialists can use this questionnaire to assess patients' anxiety levels before, during, and after ECT treatment. This assessment can help improve treatment management, provide appropriate psychological support, and reduce patient anxiety. Furthermore, this tool can enhance the treatment process and offer clearer insights into the therapy's effects.

## 2 Materials and methods

This methodological study was performed with a cross-sectional descriptive and correlation design in 2024. This was followed by extensive consultation with experts and a comprehensive review of the related literature. We chose to use the original version of the questionnaire in this study. We sent a questionnaire to the developers. We obtained permission from the authors via email to translate and use the questionnaire. The research instrument used was the ERAQ, which comprises 17 items on a 4-point Likert-type scale, including “never,” “a little,” “some,” and “quite a lot,” which quantifies the intensity of the different types of anxiety. The score for every item ranged from 1 (never) to 4 (quite a lot). It was translated and culturally adapted into Persian according to the method proposed by the WHO (World Health Organization), Wild, and Beaton in the following steps.

### 2.1 Phase I. Forward and back translations

Two faculty members who are expert translators translated the scale during the first stage of forward and backward translation. One translator had a Ph.D. in English, while another had a Ph.D. in psychiatric nursing. They both have expertise in translating specialized materials and are familiar with the concepts covered in the scale. An expert panel consisting of a psychiatrist, psychologist, mental health nurse, and research team members compared and reviewed these two translations (World Health Organization, 2009; Wild et al., 2005). This committee approved the Persian version of the ERAQ and prepared it for back-translation. A bilingual translator with expertise in both Persian and English meticulously translated the questionnaire into English. This translator had no involvement in the previous stages and had no prior exposure to the original tools, ensuring the utmost accuracy (World Health Organization, 2009).

The research team analyzed the differences between the original and translated texts, compared and contrasted them, and ultimately decided to use an English translation (World Health Organization, 2009; Wild et al., 2005). They sent it to the original developer to ensure the accuracy of the translation (Beaton et al., 2000). The comments were received, and the preliminary Persian version of the ERAQ was adjusted.

### 2.2 Phase II. Pretesting

In this phase, we administered the Persian version of the ERAQ to ten psychiatric patients who met the inclusion criteria in a pilot study to investigate its wording, scoring, and statement rating, and we modified its components (World Health Organization, 2009; Wild et al., 2005; Beaton et al., 2000). The cross-cultural adaptation of a self-administered questionnaire for use in a new cultural context does not end here, and further investigations should be conducted on the psychometric properties of the adapted measure (Beaton et al., 2000).

### 2.3 Phase III. Validity

We validated the scale regarding content, face, and construct validity, among other psychometric properties. We used the CVI (Content validity index) to ensure the best development of the questionnaire items to measure the content. By consulting a panel of 10 experts, we assessed the face and content validity of the scale using CVI methods, both qualitatively and quantitatively. The panel consisted of three psychiatrists, three clinical psychologists, two assistant professors in psychiatric nursing, and two instrument designers. We measured the CVI using four criteria: relevance, clarity, importance, and simplicity. We examined the relevance criterion at four levels: (1) irrelevant with a score of 1, (2) relevant but requiring serious adjustments, (3) relevant but requiring partial adjustments, and (4) completely relevant with a score of 4. We calculated the CVI using a formula based on expert opinion (Hyrkäs et al., 2003). According to the Using guidelines proposed by Waltz and Bausell, a CVI < 0.7 is unacceptable, a CVI between 0.7 and 0.78 requires modification and revision, and a CVI ≥ 0.79 is acceptable (Polit et al., 2007).

We utilized the item impact technique to eliminate inappropriate items and determine their relevance in the next step. Since factor analysis requires 10–15 subjects per variable, we selected 10 responders per variable to guarantee construct validity (Polit et al., 2007). The KMO (Kaiser–Meyer–Olkin) measure of sampling adequacy indicates that a KMO value of 0.6 or higher indicates the sample's adequacy (Bannigan and Watson, 2009). Since there were 17 items on the questionnaire, the sample size was considered to be *n* = 170. For ethical purposes, the respondents were informed of the research objectives and the confidentiality of the data before the sampling stage. The Ethics Committee approved the present study with the code IR.Yums.REC.1402.180. The appropriate institutional research ethics committee approved this study and conducted it according to the ethical standards outlined in the 1964 Declaration of Helsinki, its later amendments, or comparable ethical standards (Rickham, 1964). After explaining the study's purpose, we obtained all participants' written consent and verbal assent. We informed the individuals whose participation was voluntary, confidential, and anonymous. We notified them that they had the right to withdraw from the research at any time. A total of 170 hospitalized psychiatric patients with diagnoses of MDD (Major Depression Disorder), BMD (Bipolar Manic Disorder), and schizophrenia aged 20–73 years were recruited through convenience sampling and completed the 17-item EARQ. The researchers' preferences did not influence the selection of individuals with different psychiatric diagnoses. The inclusion criteria for the study were as follows: “Participants must have been willing to engage in the study.” The exclusion criteria encompassed individuals with cognitive impairments, those diagnosed with severe physical illnesses, and participants who voluntarily chose to withdraw from the study at any stage.

PCA (Principal Component Analysis) and the orthogonal rotation method (Varimax) with the maximum likelihood algorithm for factor extraction were used to conduct the EFA (Exploratory Factor Analysis) of the model. Scree plot inspection and the Kaiser criterion (eigenvalues > 1) guided the number of extracted factors. We considered factor loadings of 0.4 or higher to be significant and cross-loadings below 0.4 to be acceptable (Field et al., 2012; Hair et al., 2014). We evaluated the similarities and differences between the EFA-based modified factor structure and the original model. We used confirmatory factor analysis through maximum likelihood estimation to examine the structural validity of the scale. Essentially, the confirmatory factor analysis determined whether the questionnaire items aligned with and fit the relevant factors in line with theoretical expectations. The analysis was performed using IBM (International Business Machines Corporation) SPSS (Statistical Package for the Social Sciences) -AMOS 21 software. We used the following indices and benchmarks: normed χ^2^ (Chi) [χ^2^/df (Degree of Freedom) ≤ 2.5], RMSEA (Root Mean Square Error of Approximation) ≤ 0.07, GFI (Goodness-of-Fit Index) >0.90, CFI (Comparative Fit Index) ≥0.90, and TLI (Tucker–Lewis Index/Non-Normed Fit Index) >0.90 (Field et al., 2012; Hair et al., 2014; Ebrahimi et al., 2014). For further details, we used several key fit indices to evaluate how well our model represented the observed data, ensuring its accuracy and reliability. The normed χ^2^/df ratio of 2.1, below the recommended threshold of 2.5, indicates a strong overall fit, suggesting the model aligns well with the data without unnecessary complexity. Similarly, the RMSEA value of 0.062 is well below the cutoff of 0.07, indicating a minimal error in approximation and confirming the model's excellent fit to the data. The GFI (0.93), CFI (0.92), and TLI (0.91) all exceed the ideal threshold of 0.90, further reinforcing the idea that the model fits the data exceptionally well, explaining over 90% of the variance and offering a significant improvement over simpler models.

Although the model's fit indices demonstrate the data's validity, this study used Varimax rotation with the assumption of factor independence. This method helps to separate factors by maximizing their connections to different variables, resulting in clear and easy-to-understand outcomes. It works well when factors are independent, making it a popular choice for researchers looking for straightforward results. However, this method may also unrealistically consider relationships between factors, potentially influencing the results and affecting the construct validity.

### 2.4 Phase IV. Reliability

The questionnaire's reliability was estimated using McDonald's Omega. We performed the analysis using IBM SPSS-AMOS 22 software. We eventually sent the final report of all steps to the original developer (World Health Organization, 2009; Wild et al., 2005; Beaton et al., 2000).

## 3 Results

The present study involved 170 psychiatric patients diagnosed with MDD, BMD, and schizophrenia. The mean age of the participants was 44.1 ± 12.6 years, and 51.8% were female. Table 1 presents additional baseline characteristics of the participants.

**Table 1:** Demographic variables of participants in the study.

**Demographic variables**		**Frequency**	**Percentage**
Gender	Female	88	51.8
	Male	82	48.2
Age	< 30	28	16.5
	30–50	88	51.8
	>50	54	31.8
Marital status	Single	49	28.8
	Married	93	54.7
	Widow	28	16.5
Level of education	Illiterate	29	17.1
	Primary	30	17.6
	Guidance	31	18.2
	Secondary	48	28.2
	Bachelor's degree and higher	32	18.8
Employment status	Government job	28	16.5
	Unemployed	26	15.3
	Homemaker	63	37.1
	Self-employment	53	31.2
ECT frequency	< 5	149	87.6
	11-May	21	12.4
ECT Session	< 5	123	72.4
	10-May	47	27.6
Family support	Yes	128	75.3
	No	42	24.7

**Figure 1:**
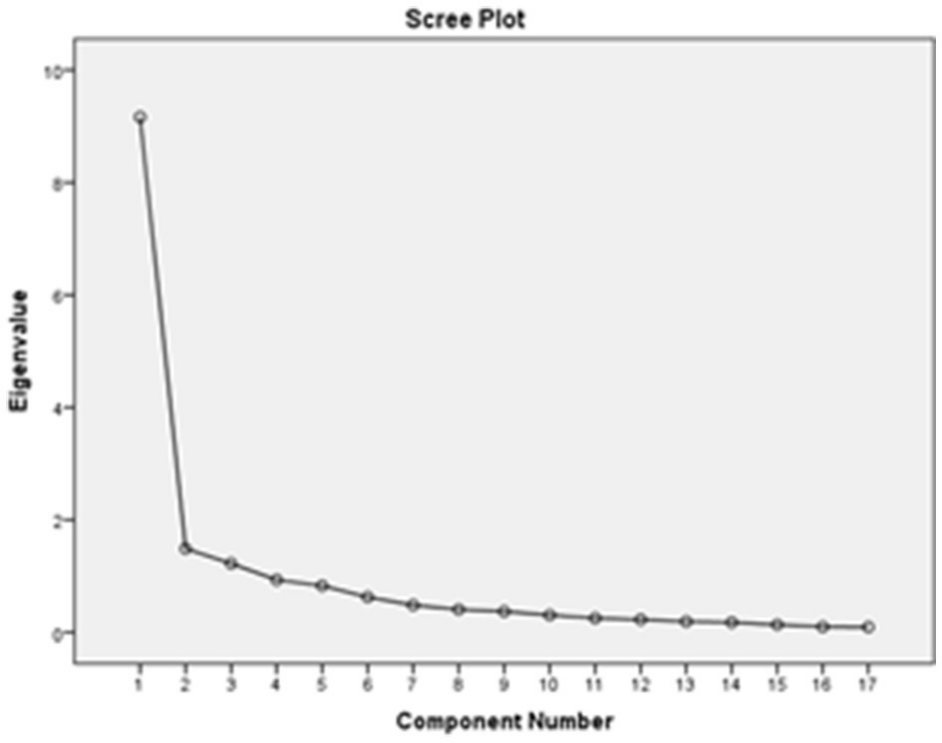
Scree plot based on factor analysis to assess the correlation between items.

We estimated the CVI of the ERAQ to be 0.97. Considering the results of the factor analysis, the ERAQ comprised three factors that confirmed the structure of the English version both in terms of the number of factors and their combination. This finding supports the construct validity of this questionnaire. Three factors were extracted using an eigenvalue >1 and a scree plot (see Figure 1). Table 2 summarizes three hidden factors with eigenvalues of 9.162, 1.489, and 1.223.

**Table 2:** Exploratory factors extracted from ERAQ.

**Factors**	**Questions**	**Factor load**	**Communalities**	**% of cumulative variance**	**Eigen value**	**McDonald's Omega**
1	I am anxious about temporary memory loss due to ECT	0.844	0.855	51.189	9.162	0.965
	I am anxious about permanent memory loss due to ECT	0.917	0.800			
	I am anxious about brain damage due to ECT	0.953	0.806			
	I am anxious about personality changes due to ECT	0.547	0.820			
2	I am anxious about having a headache after the ECT treatment	0.498	0.506	57.181	1.489	0.91
	I am anxious about the narcosis (complete anesthesia)	0.603	0.629			
	I am anxious about not waking up after the anesthesia	0.453	0.608			
	I am anxious about dying due to ECT	0.586	0.562			
	I am anxious about the use of electricity during the ECT procedure	0.979	0.877			
	I am anxious about the convulsion during the ECT procedure	0.986	0.879			
	I am anxious about what others would think of my treatment	0.390	0.559			
3	I am anxious about feeling nauseated after the ECT	0.707	0.368	64.071	1.223	0.964
	I am anxious about damage to my teeth due to ECT	0.450	0.276			
	I am anxious about the needle that is used during anesthesia	0.778	0.615			
	I am anxious about being surrendered to the medical staff during anesthesia	0.879	0.701			
	I am anxious about doing embarrassing things during anesthesia	0.389	0.542			
	I am anxious to have to wait a long time for my treatment on the morning of the ECT treatment	0.490	0.490			

Three factors were extracted that explained 64.071% of the variance in all the variables of the ERAQ. We labeled three factors, death anxiety, physical concerns, and brain concerns, according to the content of the questions. The internal consistency of the ERAQ was calculated at 0.98 using McDonald's omega. The results showed that in our study, McDonald's omega values for each factor of death anxiety, physical concerns, and brain concerns were 0.965, 0.95, and 0.964, respectively.

The KMO of sampling adequacy was 0.904, and Bartlett's test of sphericity was 2,382.476 and significant (*p* < 0.001), supporting the appropriateness of factor analysis. CFA revealed moderate fit indices to support the three subscales.

**Figure 2:**
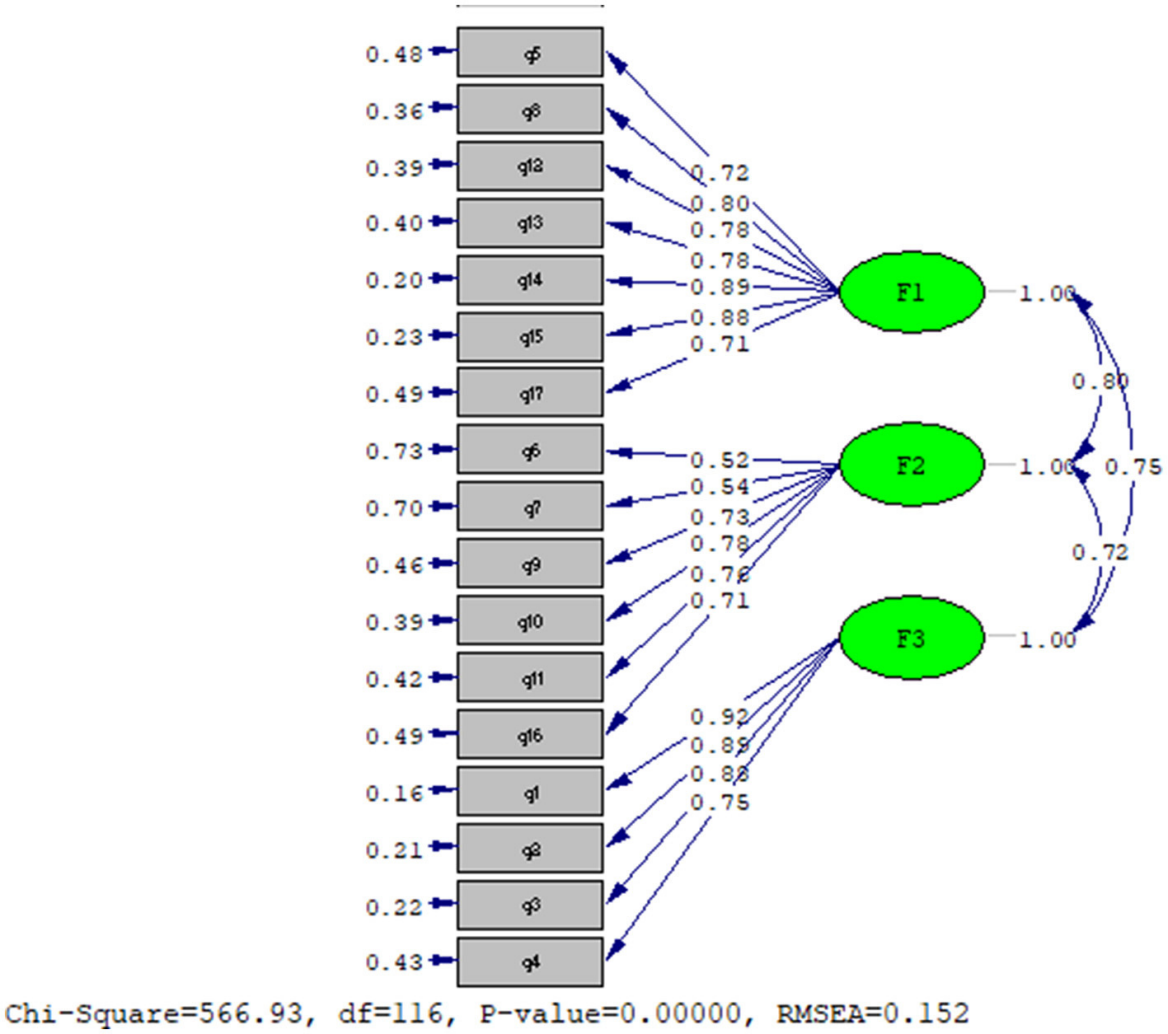
An estimated 3-factor model of electroconvulsive-related anxiety.

CMIN/DF^1^ = 4.8, *P* < 0/05, RMSEA = 0.152, CFI = 0.92, and NFI^2^ = 0.91; GFI^3^ = 0.72; DF = 116. Figure 2 shows the results that confirm the model's good fit.

## 4 Discussion

The present study aimed to assess the psychometric properties of the Iranian version of the EARQ in a sample of psychiatric patients receiving electroconvulsive therapy. This study supports the validity and reliability of the EARQ as a brief and easily administered 17-item questionnaire in a Persian Iranian population. Two-factor analyses (i.e., EFA and CFA) were used to explore the factor validity of the EARQ. Despite the English version of the EARQ indicating a single-factor structure, the present study revealed that the EARQ consisted of three factors, including brain concerns (Q1, Q2, Q3, and Q4), death anxiety (Q5, Q8, Q12, Q13, Q14, Q15, and Q17), and physical concerns (Q6, Q7, Q9, Q10, Q11, and Q16). The death anxiety score in this study was 21.2 ± 5.31, which was the most common factor of anxiety among psychiatric patients.

Obbels et al. (2020) applied IRT^4^ modeling to determine the severity of anxiety. In both the Persian and English versions of the EARQ, the overall score ranges from 17 to 68. The higher the patient's score is, the more anxiety they experience. The present study's internal consistent reliability was higher than that of the English version of the questionnaire, with a value of 0.98, calculated using McDonald's omega. In Obble's study, internal reliability was calculated using Cronbach's alpha, with a value of 0.94 (Obbels et al., 2020).

Although other studies have evaluated ECT-related anxiety in Iran, they did not use specific questionnaires (Ebrahimi et al., 2014; Ranjbar et al., 2009; Biazar et al., 2020). For instance, Ebrahimi et al. (2014) used the STAI^5^ to assess anxiety in patients after ECT (Ebrahimi et al., 2014). In line with this study, Ranjbar et al. (2009) explored the factors contributing to the fear of ECT in 200 psychiatric patients. Their comprehensive questionnaire, consisting of 28 items, identified four primary sources of anxiety: fear related to family, social, and economic concerns, fear of the treatment team's reactions, fear of the side effects of the treatment, and fear associated explicitly with the ECT method. Notably, the highest anxiety scores were attributed to fear of the ECT method, with a mean score of 23.49 ± 44.43(Ranjbar et al., 2009). This factor was the most prevalent source of anxiety among psychiatric patients in their study, whereas, in the present study, death anxiety emerged as the predominant concern. It is important to emphasize that death anxiety may vary across different communities and cultural contexts, underscoring its complex and multifaceted nature (Sharif Nia et al., 2015).

Death anxiety is a profound existential concern that significantly shapes an individual's thoughts and behavior (Sahan et al., 2018). As an inevitable and unpredictable event, death often undermines motivation and fosters pervasive fear (Fitri et al., 2020). This form of anxiety encompasses negative emotions and attitudes toward one's mortality, the deaths of significant others, or the broader concept of death itself (Abreu-Figueiredo et al., 2019). In the current study, patients experiencing death anxiety about ECT expressed a variety of concerns, most notably fear of the potential effects of anesthesia, the risk of seizures, and the anxiety surrounding how others perceive them. These patients frequently worry about losing control over their consciousness and behavior during the procedure, intensifying their overall sense of anxiety and distress.

Similarly, Biazar et al. (2020) employed a self-developed questionnaire based on Ranjbar's original instrument to assess pre-ECT anxiety in 353 patients. Their findings revealed that the most common source of anxiety was the anticipated side effects of treatment, particularly the fear of general anesthesia, which was cited by 73.2% of participants (Biazar et al., 2020). While fear of anesthesia has been recognized as a key component of death anxiety and a significant contributor to pre-ECT anxiety, Guruvaiah et al. reported that 17% of their participants experienced severe anxiety before undergoing ECT. Interestingly, their study found that patients demonstrated higher levels of anxiety related to general anesthesia than to the ECT procedure itself, suggesting that the fear of the unknown and the perceived risks of anesthesia may outweigh concerns directly associated with ECT (Guruvaiah et al., 2017).

Further qualitative research has highlighted the similarities between death anxiety in patients undergoing ECT and preoperative anxiety in surgical contexts (Frank Koopowitz et al., 2003). A study indicated that both pharmacological and non-pharmacological interventions could mitigate preoperative death anxiety, with non-pharmacological therapies being increasingly preferred due to the significant side effects often associated with pharmacological treatments. These non-pharmacological approaches, including cognitive-behavioral therapy (CBT), preoperative informational videos, hypnosis, aromatherapy, relaxation techniques, music therapy, massage therapy, and guided imagery, have been shown to significantly improve patients' emotional wellbeing and alleviate anxiety before the procedure (Wang et al., 2022).

These non-pharmacological treatments, particularly cognitive therapy, have a profound impact on reducing all aspects of ECT-related anxiety, with a specific focus on mitigating death anxiety. Other therapeutic approaches, however, are more targeted toward addressing other factors, such as brain and physical concerns (Verledens et al., 2024). Brain injury resulting from ECT can lead to notable changes in cognition and functional abilities, creating a scenario in which patients may remain physically present but experience psychological alterations (Kean, 2010). Furthermore, the brain damage associated with ECT can complicate how participants process and cope with the sense of loss, both existential and psychological, following the procedure (Shipwright and Murphy, 2024).

In another qualitative study, participants shared that their experiences with ECT led to a growing mistrust of healthcare systems, with many expressing a desire to avoid medical interventions in the future. These negative experiences with ECT resulted in both physical and psychological harm, prompting increased discussion within the medical community about the psychological trauma associated with the procedure (Johansson-Everday, 2023). This discussion highlights the need for more knowledge of the full range of physical and psychological effects of ECT on patients and the importance of combining effective therapeutic interventions to manage these outcomes.

Due to the lack of knowledge about electroconvulsive treatment's side effects, utilities, processes, etc., psychiatric patients often experience considerable anxiety (Chakrabarti et al., 2010). To address this, nurses should use the present questionnaire to assess the specific anxiety factors before and after ECT. Based on the results, they can provide reassurance and refer patients for psychological interventions, such as non-pharmacological therapies, to reduce anxiety, especially death anxiety. Additionally, decreasing waiting time for anesthesia and offering informational resources can alleviate patient concerns. The medical team's collaborative, compassionate approach will enhance patient comfort, minimize distress, and ensure a favorable treatment experience.

The strength of this study lies in the ERAQ, a concise, 17-item measure that is both specific and easy to administer. It demonstrates high reliability in assessing ECT-related anxiety among psychiatric patients. Additionally, the study's use of both exploratory and confirmatory factor analysis adds robustness, allowing for the identification of key factors contributing to this anxiety.

However, several limitations must be considered. First, although it is generally recommended to use separate samples for exploratory and confirmatory factor analyses to prevent overfitting and potential biases, we opted to conduct the CFA on the same sample due to the limited number of participants and practical constraints in recruiting additional patients. Future research with separate validation samples is essential to verify the findings from our CFA. Second, for some individuals, reading the questionnaire may result in misunderstandings, potentially affecting the accuracy of responses. For future studies, it is recommended that participant feedback be utilized to ensure a proper understanding of the questions or to establish reading and writing skills as inclusion criteria. The third limitation of this study is the use of Varimax rotation, which assumes factor independence. While this method is suitable for data with independent factors, it may not fully capture the relationships between factors, potentially affecting construct validity. Future studies should consider using oblique rotations such as Promax to achieve more accurate construct validity. Promax rotation benefits ordinal data and is widely applied in psychological and social domains, where factors are often correlated. Finally, the test's construct validity should be further examined and refined in future research to ensure the robustness and applicability of the questionnaire.

## Data Availability

The original contributions presented in the study are included in the article/supplementary material, further inquiries can be directed to the corresponding author.

## Footnotes

^1^chi-square fit statistics/degree of freedom.

^2^normed fix index.

^3^goodness-of-fit index.

^4^Item Response Theory.

^5^Spielberg State and Trait Anxiety Inventory.
